# Learning State Assessment in Online Education Based on Multiple Facial Features Detection

**DOI:** 10.1155/2022/3986470

**Published:** 2022-01-29

**Authors:** Deguang Li, Zhanyou Cui, Fukang Cao, Gaoxiang Cui, Jiaquan Shen, Yongxin Zhang

**Affiliations:** ^1^School of Information Technology, Luoyang Normal University, Luoyang 471934, China; ^2^College of Mechanical and Electrical Engineering, Zhengzhou Institute of Industrial Technology, Zhengzhou 451150, China

## Abstract

Considering that most of online training is not effectively supervised, this article presents an online leaning state assessment approach which combines blink detection, yawn detection, and head pose estimation. Blink detection is realized by computing the eye aspect ratio and the ratio of closed eye frames to the total frames per unit time to evaluate the degree of eye fatigue. Yawn detection is implemented by computing the aspect ratio of the mouth by using the feature points of the inner lip and combining it with the time of opening mouth to distinguish the mouth state. Head pose estimation is first implemented by calculating the head rotation matrix by matching the feature points of 2D face with the 3D face model and then calculating the Euler angle of the head according to the rotation matrix to evaluate the change of the head pose. Especially in yawn detection, we employ the feature points of inner lips in the calculation of the mouth aspect ratio to avoid the impact of lip thickness of various participants. Furthermore, the blink detection, yawn detection, and head pose estimation are first calculated based on the two-dimensional grayscale image of human face, which could reduce the computational complexity and improve the real-time performance of detection. Finally, combining the values of blinking, yawning, and head pose, multiple groups of experiments are carried out to assess the state of different online learners; then, the learning state is evaluated by analyzing the numerical changes of the three characteristics. Experimental results show that our approach could effectively evaluate the state of online learning and provide support for the development of online education.

## 1. Introduction

In recent years, as the online education industry evolved rapidly, many forms of education arose, establishing a dual-mode ecology of education [1]. Online learning has become extremely important due to the popularity of the Internet, especially under the effect of the novel coronavirus pneumonia (NCP) [2], and online education has ushered in an unprecedented explosive growth. Countries throughout the world are increasingly focusing on online learning and Chinese online education is also beginning to expand quickly [3, 4]. Online education has some advantages compared to traditional classroom education; the cost of online earning is extremely low, only 30%–50% of that of the classroom training, which could be carried out at any time and in any place, without being constrained by time and location; it also has the advantages of flexibility, autonomy, and personalized learning [5]. In traditional teaching, teachers and students are in a limited space, and it is easy for teachers to observe students' learning state. However, this impact has not been accomplished in the current online education. How to monitor and elevate the learning state of learners in front of the camera is a major challenge to be solved by every online course platform.

At present, many scholars have proposed specific strategies for online education [6–10], and many researches based on facial expression recognition were proposed. Sun et al. [11] designed a model of emotion calculation based on face characteristics as input data in order to assess the emotional status of learners in the current remote education system and implemented face emotion detection in the real time with SVM algorithm. Bo et al. [12] thought that the emotional state had impact on the learning effect and constructed an emotional analysis framework based on facial expression recognition in the intelligent learning environment based on the facial action coding system (FACS) proposed by the famous psychologist Ekman. Liu et al. [13] have created an intelligent environment to detect and regulate the emotions of students and carried out experiments on 3D virtual magic learning environment (3DVLE); the results of the experiments demonstrate the effectiveness and viability in promoting active learning. Sun et al. [14] applied convolutional neural network (CNN) to study how to use facial expressions to recognize emotions effectively in future online education learning. Zheng et al. [15] proposed a new pattern recognition technology to analyze learners' facial images and give tips or warnings in distance learning system. Robal et al. [16] investigated an easy approach to identify a loss of concentration in online learning by recognizing the presence or absence of a face with the activities and behaviors of MOOC users. Kong and Li [17] employed the AdaBoost face detection algorithm to detect the face region and extracted the features of the eye and mouth of online learners according to the facial expression model; then they used comprehensive strategies to evaluate the learner's condition and identified the learning state as focus, tiredness, and normal.

However, most of the above researches are based on facial expressions, while many studies have noticed that facial expressions do not play an important role in online learning, and it is not very reasonable to use basic facial expressions to evaluate the learning state of learners in the learning process. To solve the above problem, we propose a method of learning state assessment in online education based on multiple facial features detection, which mainly includes blink detection, yawn detection, and head pose estimation. In this study, blink detection is realized by computing the aspect ratio to assess the eye state and calculates the ratio of the number of closed eye frames to the total number of frames per unit time to evaluate the degree of eye fatigue. Yawn detection is implemented by computing the aspect ratio of the mouth using the feature points of inner lip and combining it with the time of opening mouth to distinguish the mouth state of open mouth, yawn, or deep yawn. In order to eliminate the influence of lip thickness on the calculation of aspect ratio of the mouth, inner lip feature points are used to avoid the interference of different mouth shape. Head pose estimation is first implemented by calculating the head rotation matrix by matching the feature points of 2D face with the 3D face model and then calculating the Euler angle of the head according to the rotation matrix to evaluate the change of the head pose. All the three detections are first calculated based on the two-dimensional grayscale image of human face, which could reduce the computational complexity and improve the real-time performance of detection. Finally, multiple groups of experiments are carried out to assess the state of different online learners, and results show that our proposed method could effectively evaluate the state of online learning and provide support and help for the development of online education.

## 2. Related Work

Most of the online education only provides learning tools such as online platforms and videos and does not have efficient monitoring mechanisms to encourage learners to attend. Many researches have tried to address this topic from various perspectives. Zhang and Duan [18] integrated face detection and image recognition to identify learning state; however, their research object only concentrated mainly on eye and mouth features without monitoring learners' head posture. Wu et al. [19] conducted a study on students' online learning focus by integrating facial expressions, eye states, and facial gestures; then student's learning focus was evaluated by this multidimensional information; however, it is not accurate to assess students' fatigue only by using the number of blinks and closed time of their eyes. Pan [20] used AdaBoost algorithm to detect learners' facial expressions, eye states, and mouth states but did not evaluate study learners' concentration by these features. Li [21] first detected the closed state of eyes and mouth and then categorized the learning state into three types, focus, normal, and fatigue, by the state data and fuzzy reasoning method, while head posture was not integrated in that study. Driver's exhaustion detection method utilizing machine learning technology has been summarized by Ngxande et al. [22]; their analyses show that support vector machine is the most frequent technology used to detect drowsiness, which is typically expressed on the face, and the tiredness degree is fully expressed on the face features including blindness, head motion, and yawn. Naz S. et al. [23] introduced an intelligent safety system that uses cameras to continuously monitor the driver; the system determines the driver's fatigue state by detecting the movement characteristics of the driver's eyes, mouth, and head and gives an alarm when necessary.

Blink detection has been widely used in many fields, such as living body detection [24] and fatigue driving detection [25]. Chau and Betke [26] presented a frame difference method for blink detection, and subsequent researchers combined frame difference blink detection and facial feature point detection realizing a highly practical living face detection system. Mandal et al. [27] proposed a driver fatigue detection system, which is composed of face detection, eye detection, and percentage of eyelid closure over the pupil over time (PERCLOS) [28], and adaptive integration algorithm is used to evaluate the degree of fatigue driving. In typical daylight conditions, Pauly and Sankar [29] suggested a method for determining sleepiness using low resolution webcam, which was built on the Haar eye-tracking cascading classifier and coupled with histogram of oriented gradients (HOG) feature and support vector machine (SVM) classifier for blink detection. Terereza [30, 31] has suggested a real-time blink detection method with the constant advancement of technology, which has demonstrated the correctness of the algorithm by extracting essential points in the eye for calculation of the aspect ratio (EAR) of the eye to perform real-time blink detection.

Yawn detection is mainly based on investigating the morphological characteristics of the mouth. Lu and Wang [32] utilized image interpolation between the two frames to find the face region and then split the face region and located the jaw and the center of the nose and then identified the driver's yawn by measuring the vertical distance between nostril midpoint and chin. Abtahi et al. [33] proposed a yawn detection method based on the geometric characteristics of the mouth, which identifies and reminds the driver of the somnolence; then many researchers [34–36] integrated blink detection and yawn detection for drowsiness detection. Omidyeganeh [37] et al. used the Viola-Jones algorithm to detect the face and mouth and then used the back-projection theory to measure the rate and number of mouth changes. Saurav et al. [38] proposed an efficient system detecting drowsiness in real time, which used the mouth aspect ratio (MAR) to represent the state of the mouth, and their system was widely used for different scenarios.

The application range of head pose is relatively wide, such as line-of-sight estimation, attention modeling, and face alignment. Fanelli et al. [39] developed a random forest-based framework to estimate the head position from the depth of the image in real time and spread it to the 3D image to identify facial features; then the voting algorithm was used on each frame separately, which could achieve real-time performance on GPU without parallel processing. Thus, the head position assessment technique based on 3D depth image [40–42] has a certain advantage, not like the approach of two-dimensional color images with insufficient precision in the assessment of head positions. Min [43] et al. proposed a facial point positioning head pose estimation approach; the facial region could be properly identified by combining the AdaBoost algorithm with the skin color model; then the head position is estimated generally with Hoff circle method and the overall accuracy is proved by experiments. Xu and Teng [44] proposed a class grading module based on the conversion of Euler angles and attention of the head; the module takes the head Euler angle of three directions as input and uses spatial information for angle correction to obtain more accurate results. Churiwala et al. [45] developed a system of drowsiness detection which mainly detects the four indicators: eye closing and blinking duration, blinking frequency, yawn, and head rotation; according to the calculation results, the fatigue degree of the driver is evaluated and audio signals are sent out when necessary to wake up the driver. Pattarapongsin et al. [46] used deep neural networks to extract facial feature points; then the head posture estimation was carried out by calculating the eye aspect ratio (EAR) and mouth aspect ratio (MAR) and 3D position was used to detect whether the driver is focused on driving.

In short, numerous researchers have employed blink detection, yawn detection, and head position estimation to investigate if attention is being concentrated, and most researches have been conducted using two popular computer vision libraries: OpenCV [47] and Dlib-ml [48]; Boyko et al. [49] analyzed and compared OpenCV and Dlib-ml on face recognition and facial feature recognition, and their experimental results proved that the two libraries have obvious advantages in the field of facial feature monitoring. Based on these two libraries, this paper studies the facial features detection and the evaluation of head posture, especially in the yawn detection; our study employs the feature points of internal lips in the calculation of the mouth aspect ratio to remove the impact of lip thickness of the various participants on the yawn detection. At the same time, to reduce the computational complexity of detection, the blink detection, yawn detection, and head pose estimation in this paper are first calculated based on the 2-dimensional grayscale image of human face; then head pose estimation is calculated by matching the 2D face feature points and 3D face model in feature point extraction, which could improve the real-time performance of detection. Finally, several groups of experiments show that the method proposed in this paper can be effectively applied to online learning state detection and provide technical assistance for online education.

## 3. Proposal and Design of Our Approach

**Figure 1:**
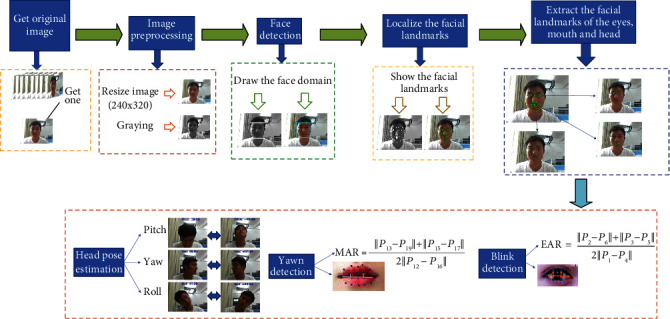
Overview of our approach.

First, we use a supervision camera for the online education to obtain a learner's learning video stream in real time and detect every frame image of the video stream and then obtain the position information of the facial features according to the detected region of the face and finally calculate the face feature values by using the position of the facial feature. Blink detection is realized by computing aspect ratio of the eyes to assess their state, and the ratio of the number of closed eye frames to the total number of frames per unit time is calculated to evaluate the degree of eye fatigue. Yawn detection is implemented by computing the aspect ratio of the mouth using the feature points of inner lips and combining it with the time of opening mouth to distinguish the mouth state of open mouth, yawn, or deep yawn. The head posture evaluation is based on the Euler angle of the head (Pitch, Yaw, and Roll). In a three-dimensional space, the Pitch (rotating around the *x*-axis) value changes significantly when nodding and looking up, the Yaw (rotating around the *y*-axis) value changes significantly when shaking your head left and right, and the Roll (rotating around the *z*-axis) value changes greatly when swinging your head left and right; these three angle values could be used to describe the changes of head posture in three-dimensional space. By combining the characteristics of blinking, yawning, and head posture, the learning state is evaluated by analyzing the numerical changes of the three characteristics; then the goal of detecting the learning state of learners is implemented. The steps of our proposed method are shown in Figure 1, and our method mainly includes face feature points detection, blink detection, yawn detection, and head pose estimation.

### 3.1. Facial Feature Points Detection

**Figure 2:**
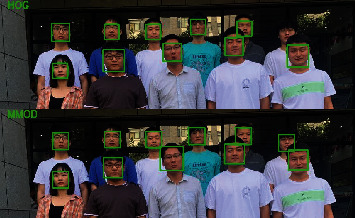
Detection results of MMOD and HOG of group 1.

Facial feature points detection first needs to obtain the location information of the face. Currently, Dlib provides two kinds of face detectors: One face detector is based on the classic histogram of oriented gradient (HOG) feature, in conjunction with linear classifier SVM, pyramid image, and sliding window detection scheme. The other is based on maximum-margin object detector (MMOD), a face detector implemented using the deep learning face detection. The HOG method simplifies the image by extracting useful information from the image and removing irrelevant information, which could guarantee fast detection speed and real-time detection; also the HOG face detector does not need to prepare a predetection model. From the point of view of detection data and detection effect, the accuracy of MMOD CNN face detection is higher than that of HOG detection, but its detection process is time-consuming and occupies serious resources; thus the real-time performance of MMOD face detector is not good. Furthermore, the detection method based on MMOD needs to load the predetection model. The detection effect and performance of the two are compared on multiple groups of detection data, as shown in Table 1 and Figure 2. Although the MMOD detection model does have higher precision, its real-time performance cannot be ensured, while the HOG based face detection method could ensure real-time detection. At the same time, as the detecting object in this study is online learning participants, there is usually a single person in the detection scenario, and the accuracy of the HOG model is also quite high in the individual person detection scene, so we use the HOG model to detect the face throughout the online learning process in real time.

**Figure 3:**
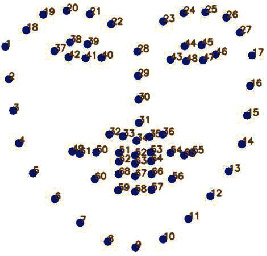
68 key facial features model of Dlib.

After detecting the face region based on HOG, the location information of the facial feature points is obtained. The 68 key facial features detection of Dlib is based on the millisecond face alignment with an ensemble of regression trees [50–52]; the face shape is turned to the real shape step by step from the current shape by building a cascade residual regression tree (GBDT). A residual regression quantity is stored on each leaf node of each GBDT; when the input falls on a node, the residual is added to the input for the purpose of regression. Finally, all residuals are superimposed together to complete the purpose of face alignment. The 68 key facial features are 17 points on the chin, 10 points on the eyebrows, 9 points on the nose, 12 points on the eyes, and 20 points on the mouth. For blinking, yawning, and head posture detection, the extracted facial points of eye, mouth, and head are prepared, and 68 facial features are indicated in Figure 3.

### 3.2. Blink Detection

**Figure 4:**
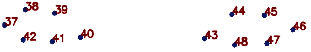
Eye feature points of 68 facial feature points model.

Eye aspect ratio (EAR) proposed by Cech and Soukupova [31] is an important metric to evaluate the state of eye; its calculation is different from that in the traditional blinking image processing method. The method first gets the position information of the eye feature points; then the aspect ratio of the eye is got by simple computation; finally, the state of the eye is evaluated by EAR, which is simpler than the traditional method.  Figure 4 shows the feature points information of the eye.

**Figure 5:**
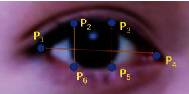
Eye feature points of the real object.

The feature points of each eye consist of 6 points, and the feature point identification number increases clockwise from the left corner of the eye. The feature points of the eyes in the real object shown in Figure 5 are marked from the number 1, which can not only clearly describe the key points of the eyes in the real object but also visually show the width and height of the eyes; among them, P1, P2, P3, P4, P5, and P6 are the six key feature points of the eye. The vertical and horizontal distances of the eyes can be calculated on the basis of position information of the feature points of the eyes; thus the aspect ratio of the eyes could be obtained according to (1) indicating the eye state. The numerator of the equation is used to calculate the distance between the longitudinal key points and the denominator is used to calculate the distance between the transverse key points, as the numerator has two sets of longitudinal key points and there is only one set of transverse key points in the denominator; the denominator has to be multiplied by 2 for the calculation of the distance of the transverse key points.

(1) $$\begin{array}{c} \text{EAR}=\frac{\left\|P_{2}-P_{6}\right\|+\left\|P_{3}-P_{5}\right\|}{2\left\|P_{1}-P_{4}\right\|}. \end{array}$$

**Figure 6:**
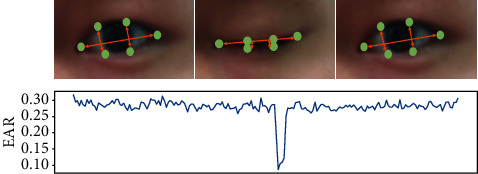
EAR changes with different eye states.

According to (1), it can be concluded that when the eyes are open, the numerator and denominator are approximately constant and the change range of EAR is small. However, when the eyes are closed, the numerator rapidly drops to zero, leading to a significant change in EAR; Figure 6 visually shows the change of EAR during blinking with real legends and curves. The equation demonstrates a fairly small quantity of computation and a relatively simple method; thus, the EAR computation does not involve too many computer resources and does not affect the detection in real time. According to the curve of EAR changing with time, the change of EAR is not obvious when the eye is open, and it drops rapidly and approaches zero when the eye is closed. When the eye is opened again, it tends to be constant again; the whole process mentioned above is a process of blinking. Therefore, the judgment of blinking only needs to introduce a threshold parameter; when the EAR is lower than the threshold, the eyes are considered to be closed or squinted; when the EAR is higher than the threshold, the eyes are judged to be open.

### 3.3. Yawn Detection

**Figure 7:**
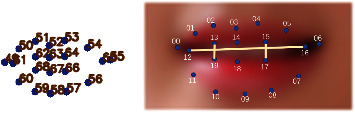
Mouth feature points of the Dlib model and real object.

Yawn is a fatigue behavior manifested by mouth; we first use Dlib to get location information from of the lip feature points of the mouth, calculate the vertical distance of the lips points according to their location and then assess the mouth state, and then distinguish the yawn state according to the duration time of mouth opening. There are 20 mouth feature points in Figure 7, among which points 49 to 60 are the outer lip feature points and points 61 to 68 are the inner lip feature points.

As shown in Figure 7, we select inner points 12, 13, 15, 16, 17, and 19 of the lip to calculate the mouth aspect ratio (MAR) and then determine the mouth state according to the value of MAR and its duration. During the opening of the mouth, the distance between the longitudinal key points in the middle of the inner lip varies greatly. Therefore, points 13 and 19 and points 15 and 17 are selected as the two groups of points to calculate the longitudinal distance, and points 12 and 16 are selected as the group of points to calculate the transverse distance; the six previously mentioned feature points are used to calculate MAR expressed in (2) to represent the opening degree of the mouth.

(2) $$\begin{array}{c} \text{MAR}=\frac{\left\|P_{13}-P_{19}\right\|+\left\|P_{15}-P_{17}\right\|}{2\left\|P_{12}-P_{16}\right\|}. \end{array}$$

Yawning should be assessed by calculating MAR and the opening time of mouth, and there are three thresholds needed to be specified, namely, the threshold for opening, threshold for yawning, and deep yawning. In this paper, the previously mentioned thresholds are determined through multiple groups of experiments, and their rationality and accuracy are verified in the experimental part. In order to quantify time, frame number is used to represent the duration in this paper, and, according to the number of opening mouth frames, the detection results are divided into open mouth (open mouth behavior such as speaking), yawning (the number of opening mouth frames more than 25 but less than 50), and deep yawning (the number of opening mouth frames more than 50 frames). In general, deep yawning is an obvious indicator of fatigue; thus it accounts for a large weight in judging user fatigue.

### 3.4. Head Pose Estimation

**Figure 8:**
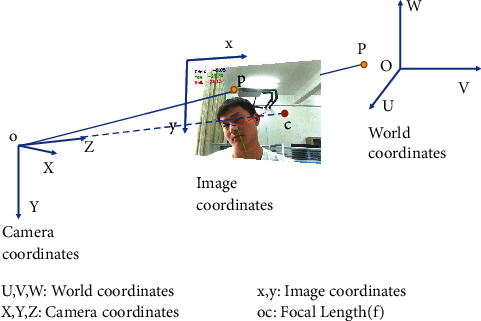
Coordinate system conversion.

Head pose estimation is usually measured by calculating head offset. In computer vision, the pose of an object refers to its position relative to the camera's position. To calculate the position information of the face in the 3D space, the feature points of the face are detected on the image in the 2D space, and the position information of these feature points is extracted; then get the head rotation matrix is got by matching the feature points of 2D face with those of the 3D face model, and finally the Euler angle of the head is calculated according to the rotation matrix; the aforementioned steps are a usual way to estimate head poses [53–57]. The attitude of an object relative to the camera can be represented by a rotation matrix and a translation matrix; the translation matrix (expressed by *T* in (3)) is the spatial position relation matrix of the object relative to the camera, and the rotation matrix (expressed by *R* in (3)) is the spatial attitude matrix of the object relative to the camera. The transformation from 2D space to 3D space requires coordinate system conversion as shown in Figure 8.

Detailed conversion relationship among the world coordinate system (UVW), camera coordinate system (XYZ), image center coordinate system (UV), and pixel coordinate system (XY) is shown in the following equations. The world coordinate system is converted into the camera coordinate system as shown in the following formula:

(3) $$\begin{array}{c} \left(\begin{array}{c} X\\ Y\\ Z \end{array}\right)=R\left(\begin{array}{c} U\\ V\\ W \end{array}\right)+T=\left[R|T\right]\left(\begin{array}{c} U\\ V\\ W\\ 1 \end{array}\right). \end{array}$$

The conversion of camera coordinate system to a pixel coordinate system is shown in the following formula:

(4) $$\begin{array}{c} s\left(\begin{array}{c} x\\ y\\ 1 \end{array}\right)=\left(\begin{array}{ccc} f_{x} & 0 & c_{x}\\ 0 & f_{y} & c_{y}\\ 0 & 0 & 1 \end{array}\right)\left(\begin{array}{c} X\\ Y\\ Z \end{array}\right). \end{array}$$

Based on the two above equations, the relationship between pixel coordinate system and world coordinate system is shown in the following equation:

(5) $$\begin{array}{c} s\left(\begin{array}{c} x\\ y\\ 1 \end{array}\right)=\left(\begin{array}{ccc} f_{x} & 0 & c_{x}\\ 0 & f_{y} & c_{y}\\ 0 & 0 & 1 \end{array}\right)\left[R|T\right]\left(\begin{array}{c} U\\ V\\ W\\ 1 \end{array}\right). \end{array}$$

Equation (5) can be solved iteratively by direct linear transform (DLT) algorithm [51] combined with least squares, and the objective function of least squares is shown in the following equation:

(6) $$\begin{array}{c} J={\left({\hat{x}}_{i}-x_{i}\right)}^{2}+{\left({\hat{y}}_{i}-y_{i}\right)}^{2}, \end{array}$$

where ${\hat{x}}_{i}$ and ${\hat{y}}_{i}$ are predicted values and the others are measured values. If the camera has radial and tangential distortion, it needs to use the image center coordinate system for transformation. The conversion of camera coordinate system to the center coordinate system is shown in the following equation:

(7) $$\begin{array}{c} \left(\begin{array}{c} u\\ v \end{array}\right)=\left(\begin{array}{c} \frac{X}{Z}\\ \frac{Y}{Z} \end{array}\right). \end{array}$$

Considering the distortion, equation (7) needs to be further converted to the form shown in the following equation:

(8) $$\begin{array}{c} u=u\left(1+k_{1}r^{2}+k_{2}r^{4}+k_{3}r^{6}\right)+2p_{1}uv+p_{2}\left(r^{2}+2u^{2}\right),\\ v=v\left(1+k_{1}r^{2}+k_{2}r^{4}+k_{3}r^{6}\right)+2p_{2}uv+p_{1}\left(r^{2}+2v^{2}\right). \end{array}$$

Finally, the image center coordinate system is converted to pixel coordinate system as shown in the following equation:

(9) $$\begin{array}{c} s\left(\begin{array}{c} x\\ y \end{array}\right)=\left(\begin{array}{ccc} f_{x} & 0 & c_{x}\\ 0 & f_{y} & c_{y} \end{array}\right)\left(\begin{array}{c} u\\ v\\ 1 \end{array}\right). \end{array}$$

According to the above equations, the rotation matrix and translation matrix can be solved by obtaining the position information of face feature points, pixel coordinates, and camera parameters in the world coordinate system; then the function provided by OpenCV is used to get the rotation matrix, and finally the Euler angle is calculated according to the rotation matrix. Usually Euler angle describes the posture of an object in a three-dimensional coordinate system; in this article, Euler angle is an important index for judging the degree of concentration of learning.

**Figure 9:**
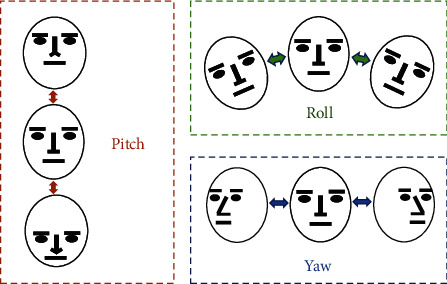
Head posture change.

Figure 9 is a schematic diagram of the Euler angle of the head. The Pitch value changes obviously when the head is nodded and raised, the Roll value changes greatly when the head is swung left and right, and the Yaw value changes significantly when the head is turned left and right. The head posture is estimated based on the real-time data of the head Euler angles, and then whether the learner is focused on learning or dozing is assessed. It can be seen from the schematic diagram that when a learner is dozing off, his head will be nodding or swinging; that is, these behaviors are accompanied by changing in the Pitch value, the Roll value, and Yaw value.

## 4. Implementation and Validation

### 4.1. Model Training

**Figure 10:**
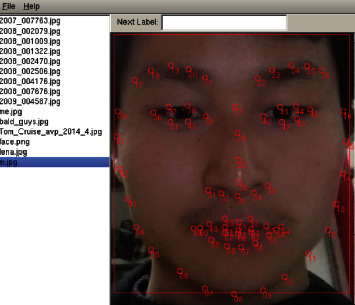
Face feature annotation.

There are two types of data sets used in the training of 68 face key points prediction model. One is the public data set named ibug [58–61], including 300-W, AFW, Helen, and LFPW; the data set contains images and annotated data. The other is the face data set without annotation data, which needs to be annotated with the help of annotation tools. Dlib provides an annotation tool, imglab, which generates the imglab.exe file after compiling imglab. Before annotation, we first create an XML file to store annotation data and then run imglab.exe to annotate the face image.  Figure 10 shows an example of annotating the face, and the annotated data is automatically saved to the selected XML file. Then our prepared data is divided into training set and test set. The XML file contains the location information of the face and the location information of 68 face key points. To train the face key point detection model, a trainer is first created to train the face key point detector, and then the training parameters are adjusted according to the actual demand. The algorithm principle of face key point detector comes from the method in the article of Kazemi and Sullivan in 2014 [52]. Key points regression is carried out through multicascade regression tree expressed as follows:

(10) $$\begin{array}{c} {\hat{s}}^{\left(t+1\right)}={\hat{s}}^{\left(t\right)\;}+r_{t}\left(I,{\hat{s}}^{\left(t\right)\;}\right), \end{array}$$

where ${\hat{s}}^{\left(t\right)\;}$ represents the shape of the *t*-th level regressor, *t* represents the number of cascaded levels, *I*  represents the image, and *r*_*t*_ represents the update amount of the *t*-th level regressor, and the update strategy used in our experiment is the gradient boosting decision tree (GBDT) [62].

**Figure 11:**
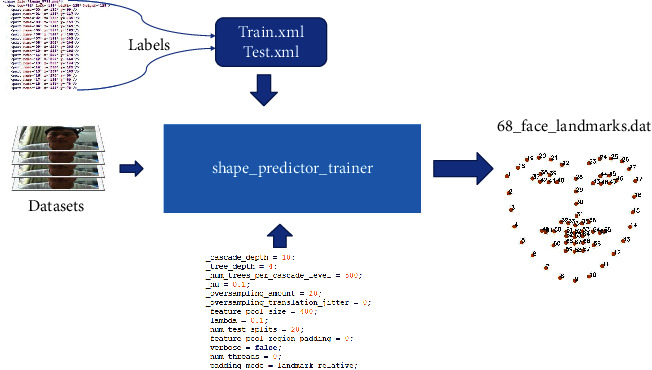
Process of model training.

Figure 11 shows the process of model training, where the training parameter cascade_depth represents the number of cascade levels, tree_depth is the depth of the tree, nu is a regular term, where its value range is (0, 1], num_trees_per_cascade_level is the number of trees contained in each level of cascade, and oversampling_amount is the multiple of random deformation of the training sample to expand the sample. Feature_pool_size pixels are randomly sampled from the images in each hierarchical link, and these pixels are used as the feature pool of the training regression tree. Such sparse sampling can ensure that the complexity of training is lower than that of training from all pixels of the original image. Of course, the larger the parameter value is, the higher the accuracy is usually, but it will also be more time-consuming. Whether to split nodes in the regression tree is determined by calculating whether the strength difference of pixel pairs meets the threshold. If the strength of the selected pixel pairs is greater than the threshold, it indicates that the nodes in the regression tree need to be split further.

### 4.2. Realization of the Detection

The main implementation process of blink detection includes the following steps. (1) Use OpenCV to call the local camera and read a frame image from the video stream. (2) According to the conversion relationship between RGB and YUV color space, establish the corresponding relationship between brightness and the three-color components; then the brightness value is used to represent the gray value of the image; thus the calculation complexity during detection can be reduced by gray processing. (3) Call the HOG face detector built in Dlib to detect the gray processed image and obtain the face position information. (4) Load the feature point prediction model and use the face key point detector to get the face feature points according to the face position information. (5) Finally, the location information of left and right eye feature points is extracted from face feature points. The EAR values of left and right eyes were calculated as well as weighted average to obtain the final EAR value, the blink is evaluated according to the EAR value and the detection results are output, and then the face rectangular box is drawn using OpenCV, the eye convex hull is calculated according to the eye key points, and the eye contour is drawn in real time according to the convex hull.

In eye detection, the first and most important point is to obtain the key points of the eye. In this paper, the EAR calculation uses the eye feature points with serial numbers 36 to 47 as shown in Figure 4, and the calculation is carried out according to (1) in Section 3.2. The second important issue is that the determination of eye opening and closing needs to determine an EAR threshold (using *α* to represent the EAR). After a large number of experimental calculations and verifications, a good detection effect is obtained when the threshold is 0.20. When *α* is less than the threshold value, eyes are considered as closed; when *α* is greater than the threshold, the eyes are considered to be open, and the eye state is expressed by (11), where *E* represents the state of the eye; when the value of *E* is equal to 1, indicating that eyes are open, the state result is OPEN; when the value of *E* is 0, the eyes are closed, and the display state is CLOSE.

(11) $$\begin{array}{c} E=\left\{\begin{array}{cc} 1, & \alpha \geq 0.20,\\ 0, & \alpha <0.20. \end{array}\right. \end{array}$$

**Figure 12:**
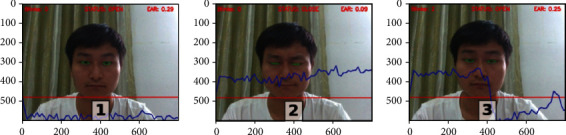
Different eye states.

From Figure 12, the eyes in Figure 1 are open, the eyes in Figure 2 are closed, and the eyes in Figure 3 are changed from closed to open. The whole process of blinking is shown in Figure 3; combining equation (11), it can be known that when the eye state is open, *E* is 1 and *α* ≥ 0.20, indicating that the eyes are open. When the eye is close, *E* is 0 and *α* < 0.20, indicating that the eyes are closed. A blink action is completed through open-close-open steps, and the status parameter STATUE will not change when the eye remains open or closed.

The core of yawn detection is to extract effective mouth feature points to calculate the aspect ratio of the mouth (MAR) and find the threshold and duration of MAR to evaluate the state of the mouth. The first four steps of yawning detection are the same as those of blink detection; the next step is to extract mouth feature points numbered 49 to 68 as shown in Figure 7. However, in order to eliminate the influence of lip thickness on the calculation of MAR, feature points numbered 62, 68, 64, 66, 61, and 65 of inner lips are selected to calculate the aspect ratio of the mouth as shown in equation (2). Then, compared with the defined threshold of MAR and combined with the duration of MAR, the mouth state is evaluated. Finally, the convex hull profile is drawn based on the feature points of the inner lips.

**Figure 13:**
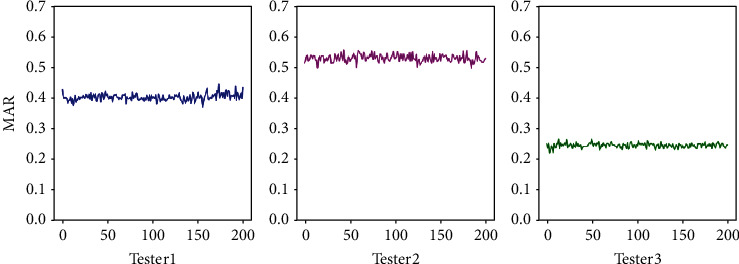
MAR of different people without mouth opening.

In the experiment, using some inner lip feature points to calculate MAR could avoid the interference of mouth shape. Due to the differences of mouth shape among different people, some researchers use outer feature points of the mouth to calculate MAR, but the MAR values of different people are different.  Figure 13 shows the change curves of MAR of three testers with different mouth shapes before opening their mouths; the tester in the middle curve has a large lip thickness; thus the shape of mouth has a great influence on MAR calculation. To solve the problems, this paper extracted the feature points of the inner lip in the experiment to calculate MAR; the specific method is to further extract the feature points of the mouth to obtain a subset of feature points (numbered as 62, 68, 64, 66, 61, and 65 in Figure 7). Based on this group of feature points, the convex hull is obtained and the contour of the inner lip is drawn. Furthermore, MAR calculation for participants with different mouth shapes could achieve similar values under the condition of mouth closure, thus eliminating the influence of mouth thickness on the calculation of aspect ratio of mouth.

Through a large number of experiments, the optimal value of the final opening threshold is set to 0.35. As shown in equation (12), *M* represents the state of the mouth;  *β* represents the value of MAR; when *β* ≥ 0.35, it means the mouth is in the open state; when  *β* < 0.35, it means the mouth is closed.

(12) $$\begin{array}{c} M=\left\{\begin{array}{cc} 1, & \beta \geq 0.35,\\ 0, & \beta <0.35. \end{array}\right. \end{array}$$

**Figure 14:**
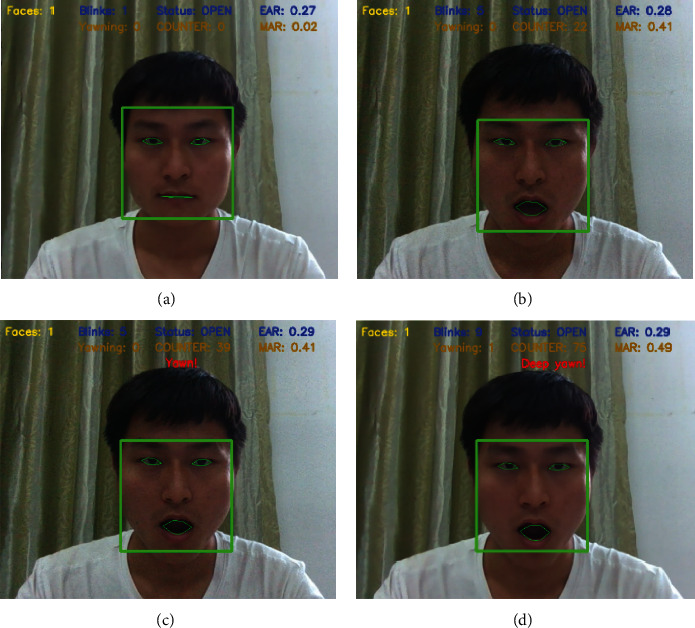
Different mouth states. (a) No open mouth. (b) Normal open mouth. (c) Yawn. (d) Deep yawn.

Yawn assessment should consider nonyawn mouth opening behaviors, such as speaking and eating snacks. These mouth opening behaviors could be distinguished by using the above threshold. In order to ensure the accuracy of yawning evaluation, the duration of mouth opening shall be considered. Based on the duration, the open state of mouth is divided into general mouth opening, yawn, and deep yawn, as shown in the following formula:

(13) $$\begin{array}{c} Y=\left\{\begin{array}{cc} 0, & \text{COUNTER}\in \left(0,25\right),\\ 1, & \text{COUNTER}\in \left[25,50\right],\\ 2, & \text{COUNTER}>50, \end{array}\right. \end{array}$$

where *Y* is the state when the mouth is open and COUNTER is the frame counter. After a lot of experiments, the yawn frame threshold is set to 25 and the deep yawn frame threshold is set to 50. Here, the total number of frames COUNTER represents the duration of mouth opening; the larger the frame value, the longer the duration of mouth opening. When COUNTER is 0∼25, the value of  *Y* is 0, indicating that the mouth is generally open (e.g., speaking, singing, etc.); when the value of COUNTER is 25∼50, the value of *Y* is 1, and the mouth state is yawn; when the value of COUNTER is more than 50, the value of *Y* is 2, indicating that the mouth is in deep yawn state. The experimental results are shown in Figure 14, in which different detection results are presented according to the value of frame counter. The MAR value tends to 0 under normal conditions; when the value of COUNTER is less than 25, it is assessed as general open mouth behavior; when the value of COUNTER is between 25 and 50, it is considered to be in yawn state, and yawn hint is given in this case; when the value of COUNTER exceeds 50, the detection result presents the hint of deep yawn.

**Figure 15:**
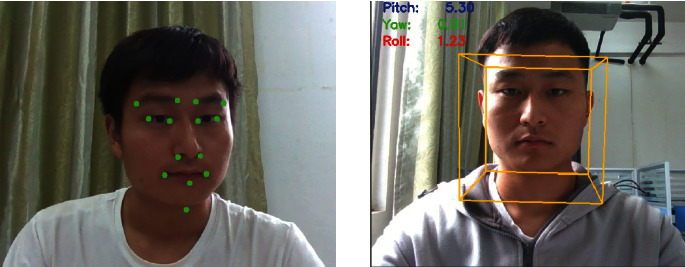
Feature points of the head and the 12-axis cube of head.

The first four steps of head pose estimation are the same as those of the previous detection; the next step is to extract facial feature points: left eyebrow left point 17, left eyebrow right point 21, right eyebrow left point 22, right eyebrow right point 26, left eye left point 36, left eye right point 39, right eye left point 42, right eye right point 45, nose lower left point 31, nose lower right point 39, mouth left outer point 48, mouth right outer point 54, mouth center lower point 57, and chin lowest point 8, as shown on the left sides of Figure 15 and Figure 3. The function of solvePnP provided by OpenCV is used to calculate the rotation matrix and translation matrix, the rotation matrix is converted into a rotation vector according to the Rodrigues formula, and then the Euler angles (Pitch, Yaw, and Roll) are calculated; the calculated Euler angles are output (results are retained two decimal places) and a cube with 12 axes is drawn based on facial feature points to achieve a 3-dimensional vision shown on the right in Figure 15, which could visually identify the head posture.

Head pose not only reflects the fatigue of learners but also reflects their concentration on learning. In this paper, Pitch and Roll are used to evaluate the sleepiness of learners, and Yaw is used to estimate the concentration of learners. Pitch, Yaw, and Roll are three rotation angle parameters of Euler angle. Pitch value changes greatly when head is nodded up and down, Yaw value changes obviously when head is turned left and right, and Roll value changes obviously when head is swung left and right. According to experimental verification, the threshold value of Euler angle is ±15; that is, the range of Euler angle in normal state is −15∼15, expressed by the following formula:

(14) $$\begin{array}{c} H=\left\{\begin{array}{cc} 1, & \text{Pitch}\in \left[-15,15\right]\text{ or Yaw}\in \left[-15,15\right]\text{ or Roll}\in \left[-15,15\right],\\ 0, & \text{otherwise,} \end{array}\right. \end{array}$$

where *H* is the state of the head pose. If the head pose is in the normal state, its value is 1, and the value is 0 when the head pose is in an abnormal state. The behaviors in the abnormal state include deviation of the sight and dozing off. Three frame counters PCOUNTER, YCOUNTER, and RCOUNTER need to be introduced to evaluate the deviation of sight and doze behavior; these three frame counters correspond to Pitch, Yaw, and Roll, respectively, and are used to record the number of consecutive frames of different behaviors of the head pose, expressed by the following formula:

(15) $$\begin{array}{c} P=\left\{\begin{array}{cc} 1, & \text{PCOUNTER}>60\text{ or RCOUNTER}>60,\\ 2, & \text{ YCOUNTER}>60. \end{array}\right. \end{array}$$

where *P* is the head pose detection result when the value of *H* is zero. When frame counter PCOUNTER of Pitch or frame counter RCOUNTER of Roll is greater than 60, it is considered as fatigue state. When Yaw's frame counter YCOUNTER is greater than 60, it is considered to be in a line-of-sight state.

**Figure 16:**
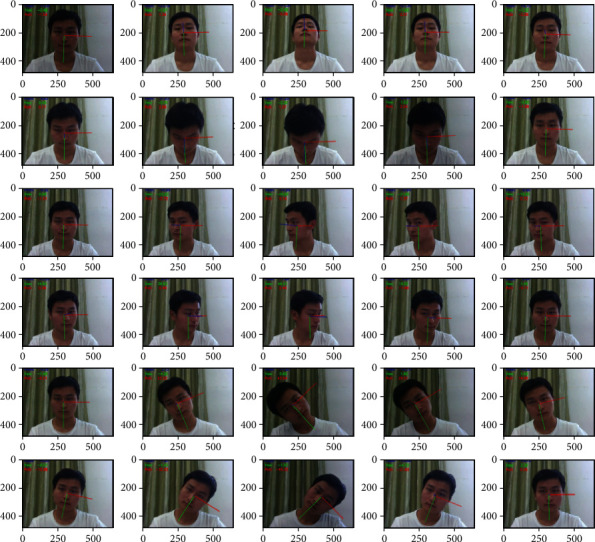
Head pose detection.

**Figure 17:**
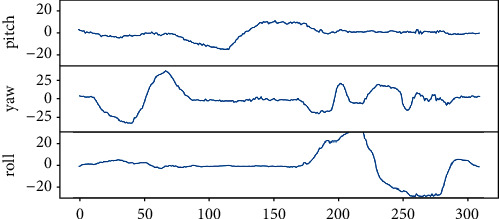
Changes in various dimensions of Euler angle of head.

The first two lines of Figure 16 show 10 extracted frames from the video stream that detects Pitch behavior, recording the whole process of nodding up and down. The last two lines are 10 frames taken from a video stream that detects Roll behavior, recording the entire process of the left and right head. The middle two lines of images detect Yaw's behavior, recording the whole process of the left and right head turn. The three curves in Figure 17 record data changes in three directions of Euler angle of the head. Pitch curve reflects the change of Pitch value; when Pitch is not in the range of −15∼15, PCOUNTER starts counting, and the other curves are the same as the Pitch curve.

## 5. Results and Discussion

Previous studies have shown that normal blink rates range from 10 to 15 blinks per minute. When learners are in the state of concentration, the number of blinks is significantly reduced, and the blinking frequency decreases. When they are in fatigue, their eyes will remain closed for a period of time, and their blink rate continues to decrease, even to zero. Therefore, it is not reasonable to only use blink rate to assess the fatigue degree. In this paper, eye closing rate is used to measure eye fatigue, eye closing rate refers to the ratio of the number of closed frames to the total frames per unit time, the threshold is set to 0.20, and if the eye closing rate is greater than 0.20, the learner's eyes are considered to be in a state of fatigue. When the learner is tired, not only the eyes but also the mouth shows fatigue behavior; thus yawn could also be used to assess the learner's fatigue degree. When the learner's eyes and mouth are in fatigue state, the learner's state could be evaluated more accurately if the learner's head pose is considered comprehensively. In general, learners' sight direction is determined by head pose; Pitch and Roll values of Euler angle of the head show drowsiness characteristics through head pose. At the same time, when Yaw value of Euler angle of the head is in an abnormal range, learners' sight deviates and their learning concentration is not high.

**Figure 18:**
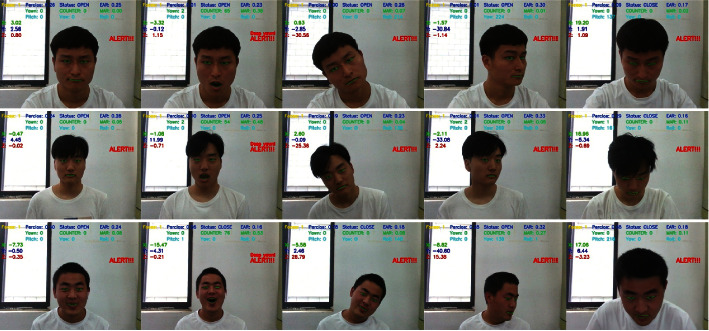
State detection of different testers.

In our experiment, participants' characteristic values of eye closing rate, yawn, and Euler angle (Yaw, Pitch, and Roll) are combined comprehensively to assess the learning status of learners. In order to accurately get the warning thresholds of the three characteristic values, this paper designed multiple groups of experiments of learning state detection involving multiple groups of students in different periods as shown in Figure 18. The detection period used in this paper is one minute; within one minute, if the learner's eye closure rate is greater than 0.2, or the learner yawns twice (or yawns deeply once), or the head Euler angle is not within the threshold and exceeds 120 frames, an alert will be given to the learner.

Figure 18 shows the detection on different testers; the first column in the figure indicates that the eye closing rate has exceeded the normal range and the patient is in a fatigue state. In the second column, yawn reaches the warning value. In the third column of the figure, Roll value changes significantly when the head is moved left or right. In the online learning scenario, Roll value reflects the sleepiness degree of the learner. The Roll value in the figure is not in the normal range and has exceeded 120 frames, indicating that the learner is becoming sleepy. The fourth column in the figure is the simulated scene of learners dozing off. According to the action of nodding, the Pitch value will change correspondingly. If the Pitch value exceeds the threshold and lasts for 120 frames, a hint will be given. The fifth column in the figure represents the detection results of learners turning their heads to the left or right. If the Yaw value is between −15 and 15, it is considered normal learning and there will be no warning. If the Yaw value is beyond the threshold range and greater than 120 frames, it is sight deviation and there will be a warning.  Table 2 list some experimental data. It can be seen that when the learner's eye closing rate exceeds 0.20 or the learner yawns twice or deeply yawns once and head Euler angle exceeds the threshold, a warning will be given.

**Figure 19:**
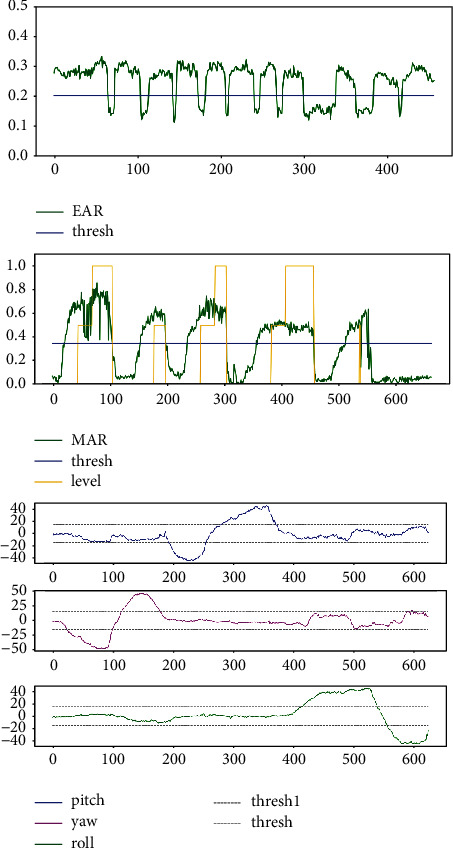
Changes of EAR, MAR, and Euler angle in a period.

Figure 19 shows some data of the learner in the process of detection. The blue horizontal line in the figure above represents eyes closing threshold, the curve of the horizontal lines above part means that eyes are in open state, and under the horizontal line means that eyes are in close state; a large variation in the curve means an occurrence of a blinking action (open-close-open). The blue line in the middle figure is the mouth opening threshold, the green curve represents the change of MAR, and the yellow line represents the occurrence of open mouth, yawn, and deep yawn. The gray line in the below figure is the threshold value of head movement. The blue curve changes greatly when the head is lowered and raised, the purple curve changes significantly when the head is turned left and right, and the green curve changes significantly when the head is moved left and right. Thus, we could assess the learner's status by these data. Based on the above experiments and results, our method is effective in assessing the state of the online learner. Some parameters in the experiment are verified by a large number of experiments, and parameters could be adjusted appropriately under different circumstances; for example, in the dark environment, the detection effect may not be very good, but better detection effect could be achieved by adjusting some relevant parameters.

## 6. Conclusion

We propose an online learning state detection method based on multiple facial features. The method could detect the state of eyes and mouth through real-time calculation and by combination with the estimation of head pose, so as to improve the accuracy of assessing the learning state. In the practical application, the online learners' learning state detection based on this method effectively could evaluate the state of online learning and provide support for the development of online education.

## Figures and Tables

**Table 1:** Comparison of HOG and MMOD model on different groups.

Image size (pix)	Model	Actual faces	Detected faces	Detection time (s)
739 × 489	HOG	11	10	0.1805
	MMOD		11	39.4186

1389 × 687	HOG	10	10	0.5066
	MMOD		10	98.5127

998 × 532	HOG	11	10	0.3560
	MMOD		11	55.0837

867 × 515	HOG	12	10	0.2384
	MMOD		11	47.5020

**Table 2:** Different detection results of our experiment.

Number	Face	Eye closing rate	Yawn	Deep yawn	Yaw		Pitch		Roll		Alert
1	1	0.23	0	0	−8.19	06	−3.48	0	−1.67	0	Y
2	1	0.03	0	1	2.00	0	1.78	0	0.73	0	Y
3	0	—	—	—	—	—	—	—	—	—	N
4	1	0.11	2	0	19.13	7	−6.32	0	4.05	0	Y
5	1	0.06	1	0	32.60	206	−19.13	35	1.70	0	Y
6	1	0.09	0	0	6.57	0	−3.43	0	−3.70	0	N
7	1	0.32	0	2	18.49	17	−13.46	0	−20.95	72	Y
8	1	0.14	1	0	4.36	0	−20.31	137	−0.59	0	Y
9	1	0.16	1	0	23.72	12	−9.98	0	8.31	0	N
10	1	0.05	0	0	2.95	0	−5.93	0	−23.53	139	Y

## Data Availability

The data used to support the findings of this study are available from the corresponding author upon request.
